# An Immunohistochemical Study of the PTEN/AKT Pathway Involvement in Canine and Feline Mammary Tumors

**DOI:** 10.3390/ani11020365

**Published:** 2021-02-01

**Authors:** Pietro Asproni, Francesca Millanta, Lorenzo Ressel, Fabio Podestà, Francesca Parisi, Iacopo Vannozzi, Alessandro Poli

**Affiliations:** 1 Research Institute in Semiochemistry and Applied Ethology (IRSEA), 84400 Apt, France; p.asproni@group-irsea.com; 2 Department of Veterinary Sciences, University of Pisa, Viale delle Piagge n. 2, 56124 Pisa, Italy; francesca.millanta@unipi.it (F.M.); fabio2463@gmail.com (F.P.); francesca.parisi@vet.unipi.it (F.P.); iacopo.vanozzi@unipi.it (I.V.); 3 Department of Anatomy Physiology and Pathology, School of Veterinary Science, University of Liverpool, Liverpool L69 3BX, UK; l.ressel@liverpool.ac.uk

**Keywords:** cat, dog, immunohistochemistry, mammary tumors, phospho-AKT, PTEN, Rictor

## Abstract

**Simple Summary:**

The PTEN/AKT pathway is involved in several human and animal tumors’ pathogenesis. This study investigates the PTEN/AKT pathway’s biological and prognostic values in canine and feline mammary tumors. PTEN, phospho-AKT (p-AKT) and Rictor expression was determined by immunohistochemistry in canine mammary adenomas and carcinomas and feline mammary carcinomas. In mammary tumors of both species p-Akt was inversely correlated with PTEN expression and positively with Rictor expression; p-Akt and Rictor expression correlated with poorer prognosis. This data could provide a rationale for further studies of this pathway in veterinary oncology due to prognostic and therapeutic implications.

**Abstract:**

Phosphatase and tensin homolog deleted on chromosome10 (PTEN), phospho-v-Akt murine thymoma viral oncogene homolog (AKT), and the Rapamycin-Insensitive Companion of mTOR (Rictor) expression was investigated by immunohistochemistry in 10 canine mammary adenomas (CMAs), 40 canine mammary carcinomas (CMCs), and 30 feline mammary carcinomas (FMCs). All the CMAs, 25 of 40 CMCs (63%) and 7 of 30 FMCs (23%), were PTEN-positive. In dogs, no CMAs and 15 of 25 CMCs (37%) expressed phospho-AKT (p-AKT), while 24 of 30 FMCs (82%) were p-AKT-positive. One of 10 CMAs (10%), 24 of 40 CMCs (60%) and 20 of 30 FMCs (67%) were Rictor-positive. In the dog, PTEN expression correlated with less aggressive tumors, absence of lymphatic invasion, and longer survival. P-AKT expression correlated with more aggressive subtype, lymphatic invasion, and poorer survival and Rictor expression with lymphatic invasion. In cats, PTEN correlated with less aggressive carcinomas, absence of lymphatic invasion, and better survival. P-AKT and Rictor expression correlated with poorer survival. PTEN expression was inversely correlated with p-AKT and Rictor in both species, while p-AKT positively correlated with Rictor expression. A strong PTEN/AKT pathway involvement in behavior worsening of CMT and FMTs is demonstrated, providing a rationale for further studies of this pathway in veterinary oncology.

## 1. Introduction

**Figure 1:**
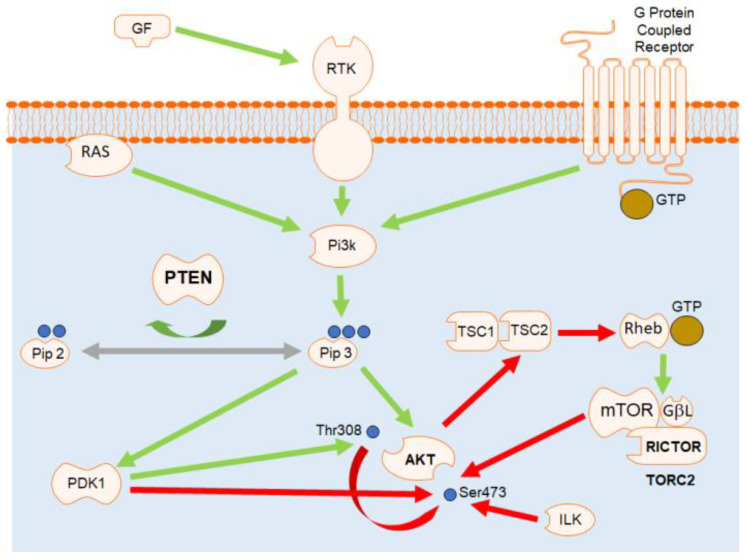
The PI3-K/Akt/mTOR signaling cascade. Stimulation of receptor tyrosine kinases (RTK) and G-protein-coupled receptors (GPCR) leads to activation of PI3-K, which then synthesizes PIP3 in the membrane. Subsequently, Akt is recruited to the membrane and phosphorylated by PDK-1 and mTORC2, modified from Hu et al., 2020 [4].

When phosphatidylinositol-3 kinase (PI3K) is activated by tyrosine kinase receptors, it phosphorylates PIP2 to generate PIP3 and activates the AKT signaling pathway. Phosphatase and tensin homolog deleted on chromosome 10 (PTEN) dephosphorylates PIP3 to PIP2 and negatively regulates the pathway. v-Akt murine thymoma viral oncogene homolog (AKT) is activated downstream of PIP3 and mediates physiological processes [1]. Mammalian Target of Rapamycin (mTOR) plays a vital role in phosphorylating AKT on threonine 308 by the mTOR kinase from the mTOR complex 2 (mTORC2) [2]. Meanwhile, the Rapamycin-Insensitive Companion of mTOR (RICTOR) is a key component of mTORC2, and it is required for mTORC2 function as demonstrated by significant inhibition of the activation of AKT by RCTOR knockdown [3]. A schematic illustration of the PI3-K/Akt/mTOR signaling pathway is shown in Figure 1.

This complex system plays a crucial role in multiple biological functions, such as cellular proliferation, survival, and metabolism [3]. This pathway is strongly implicated in tumorigenesis, and its dysregulation is associated with several human malignancies [5]. This signaling system contributes to tumor growth through the inhibition of apoptosis [3], and the stimulation of cell cycle [3] and neoangiogenesis [6]. The PTEN/AKT pathway also enhances tumor metastatic capability throughout the activation of the metalloproteinases 2 and 9 [3] and inhibition of cell adhesion molecules, such as E-cadherin protein [7].

Within this system, PTEN protein plays a crucial gatekeeper action by antagonizing the activity of phosphatidylinositol 3 kinase (PI3K) and reducing the energy available for the full activation of the pathway [8]. The lack or the reduction of PTEN expression has been reported in melanoma [9] and in breast [10], hepatocellular [11], lymphoid [12], endometrial [13], thyroid [14], and squamous cell cancer [15] in humans. In veterinary medicine, alterations in PTEN expression has been investigated in canine melanoma [16], hemangiosarcoma [17], osteosarcoma [18,19], prostate cancer [20], and in canine mammary tumors (CMTs) and feline mammary carcinomas (FMCs) [21,22,23,24] confirming PTEN tumor-suppressor role among different species and neoplasms.

AKT is the key-molecule of this signaling system, and once it is fully activated, it induces biological effects increasing tumor aggressiveness [1,25]. Among the epithelial tumors, AKT overexpression has been associated with gastric [26], squamous cell [27], pancreatic [28], ovarian [29], breast [30], and colorectal [31] carcinomas. AKT expression alterations have also been reported even in high-grade gliomas, glioblastomas, and gliosarcomas [32,33]. In veterinary oncology, AKT expression have been reported in FMTs [22] in canine hemangiosarcoma [17,34], osteosarcoma [18], melanoma [35,36], neuroepithelial [37], and perianal [38] tumors, squamous cell carcinoma [39,40], and mast cell tumors [41]. As in humans, AKT altered expression is linked to worse tumor behavior in canine and feline species.

To be activated, AKT needs phosphorylation at Threonine 308, followed by Serine 473 [25]. This last phosphorylation leads to AKT full activation mediated by the mTORC2 protein complex [25]. mTORC2 most important components are mTOR, Rictor, SIN1, mLST8, PRR5/Proctor, PRR5L15-17, and DEPTOR [42]. Among them, Rictor has been reported to play the most crucial role in the stability and integrity of the complex [43]. Rictor is implicated in human breast [44,45], prostatic [46], ovarian [47], colorectal [48] cancers and astrocytoma and gliomas [49,50]. No studies are available in veterinary medicine about Rictor involvement in neoplastic diseases.

Due to the several pieces of evidence that confirm the role of the PTEN/AKT pathway in various malignancies, including breast cancer, this study aimed to investigate its involvement in CMTs and FMCs by immunohistochemical analysis of PTEN, phospho-AKT (p-AKT), and Rictor expression. The purpose of our study is to investigate this pathway in these species and to evaluate the prognostic value of p-AKT and Rictor expression in these tumors.

## 2. Materials and Methods

### 2.1. Animals and Procedures

This study was performed at the Veterinary Teaching Hospital of the Department of Veterinary Sciences, University of Pisa, Italy, between January 2010 and December 2015. Bitches and queens submitted to mastectomy and without distant metastases, and any co-morbidity that could significantly influence cancer-specific overall survival were included. Subjects were submitted to an accurate physical examination, and a complete anamnesis was recorded. An abdominal ultrasound examination and a complete radiographic study of the thorax for the subjects’ clinical stage. Biochemical analysis and coagulation tests were performed to investigate comorbidities’ presence and evaluate patient suitability for mammary surgery a complete blood count. Owners were contacted to monitor the subjects regularly for recurrence or metastasis (once every six months) for the study period. If necessary, the subjects were submitted to a clinical check-up at the Veterinary Teaching Hospital or provided by the referring clinicians. When thoracic radiographs did not allow detecting a clear metastatic spread, the definitive diagnosis of the cause of death was made by necropsy at the Department of Veterinary Science. In some cases, death was not related to the neoplastic mammary disease, and therefore, these animals were censored at the date of the death. Finally, fifty bitches and 30 queens (mean age = 10.7 ± 1.7) met all the criteria and were included in the study. The age of examined bitches was 9.4 ± 1.9 years (range 4–14 years), and twenty-nine were purebred and twenty-one mix breed. The most represented breeds were Boxer (5), Épagneul Breton (4), German shepherd (3), Rottweiler (3), Dobermann (3), English setter (2), Miniature Poodle (2), Labrador retriever (2), Yorkshire terrier (2), Drahthaar (2) and Border collie (1). Forty-six dogs were intact females, and only four bitches were spayed at the time of mammary gland tumor diagnosis. The examined queens’ age was 10.7 ± 1.7 (range 7–14 years), twenty-four were European shorthair cats, four Persian cats, and two Siamese cats. All the queens were spayed at the time of mammary gland tumor diagnosis.

### 2.2. Tissue Processing and Histopathology

Tumor and lymph node tissue samples were fixed in 10% neutral buffered formalin, routinely processed, embedded in paraffin, and 4µm sectioned for histopathological examination. Three/four hematoxylin and eosin (HE) stained sections per nodules were investigated under the blinded condition for the clinical data to determine the histological classification according to current tumor classification [51] bitches and queens. According to the revised Elston and Ellis grading system, tumor grading was performed, as previously described for bitches [52] and queens [53]. When dogs were presented with multiple neoplastic lesions, the tumor with the most aggressive clinical and histopathological features (larger size, infiltrative growth, and undifferentiated histology) was selected for inclusion in the study [54]. Mitotic index [number of mitoses/10 high power (400×) fields, HPF], presence of lymphatic invasion, and of randomly distributed areas of necrosis within the neoplasms were also recorded for each tumor [51].

### 2.3. Immunohistochemistry

For immunohistochemistry (IHC), 4-µm-thick sample sections were mounted on Superfrost Plus slides (Thermo Scientific, Menzel GmbH & Co., KG, Braunschweig, Germany). Sections were dewaxed in xylene and rehydrated through a graded series of alcohols and deionized water. Antigen retrieval was performed with a citrate buffer (pH 6.0) in a microwave oven with a cycle of 4 min at 350 watts followed by a cycle of 15 min at 650 watts and then cooled at room temperature for 20 min. Endogenous peroxidases were blocked with Dako Real Peroxidase-Blocking Solution (Dako, Glostrup, Denmark, cod. S2023) for 10 min, then three washes with 0.05% Tween-Tris-buffered saline solution (TBST) at pH 7.6 were performed. Non-specific reactions were blocked by incubation with Ultra-V-Block solution (prediluted, Thermo, Fremont, CA, USA, cod. TA060PBQ) for 10 min. After three washes in TBST, sections were incubated for 1 h at room temperature with primary antibodies: anti-PTEN (mouse monoclonal, clone A2B1, diluted 1:50, Santa Cruz Biotechnologies, Santa Cruz, CA, USA, cod. SC-7974), anti-phospho-AKT (p-AKT) Serine473 (rabbit polyclonal, diluted 1:50, Abcam, Cambridge, UK, ab8932) and anti-Rictor (goat polyclonal, diluted 1:300, Abnova, Taipei, Taiwan, cod. PAB6780). After three washes, the sections were incubated with a biotinylated pan-specific secondary antibody (horse, diluted 1 drop per mL, Vector Labs, Inc., Burlingame, CA, USA, cod. BP-1400-50) for 30 min. After three washes with TBST, a streptavidin-peroxidase solution (prediluted, Thermo, Fremont, CA, USA, cod. N100) was placed on the slides, followed by three washes in TBST. Diaminobenzidine (Impact DAB, Vector Labs, SK-4105) was used to develop the peroxidase reaction for 10 min, and then a wash with deionized water was performed. Samples were then counterstained in hematoxylin and dehydrated through a graded series of alcohols, placed in xylene, and mounted.

The manufacturer’s datasheet provided the cross reactivity with canine tissue for the anti-PTEN monoclonal antibody and the anti-Rictor goat polyclonal antibody. Canine tissues show cross-reactivity of anti-p-AKT rabbit-polyclonal antibody [55]. Feline tissues show cross-reactivity for anti-PTEN monoclonal antibody and anti-p-AKT rabbit-polyclonal antibody [22]. IHC assay’s specificity was confirmed by omitting the primary antibody and replacing it with species-matched unrelated primary antibodies. As PTEN positive controls. Canine and feline renal glomeruli were used in each experiment, and vascular endothelium was used as an internal positive control in each slide [16]. Human breast cancer tissue sections (kindly provided by Dr. P. Viacava) were used as positive controls for phospho-AKT and Rictor IHC. For all three markers, samples were scored using a modified semiquantitative scoring system (range 0–7; positivity ≥ 3), as previously described in human mammary tumors [56] and FMTs [22]. For all the markers, cytoplasmic staining only was considered to assess the IHC scoring [21,22].

### 2.4. Statistical Analysis

Statistical analysis was performed using the statistical package SPSS Advanced Statistic 21 (SPSS Inc, Chicago, IL, USA). The correlations among PTEN, phospho-AKT, and Rictor expression, and between the three markers and individual tumor features were determined using the Fisher test. Statistical significance was based on a 5% (0.05) significance level. Cancer-specific overall survival analysis was performed using the Kaplan–Meier method and both the Tarone–Ware and the log-rank (Mantel–Cox) tests were used to investigate the relationship between different parameters and cancer-specific overall survival.

## 3. Results

### 3.1. General Data

Ten of the 50 CMTs (20%) were diagnosed as adenomas and 40/50 (80%) as carcinomas (CMCs). All the adenomas were of simple type, while the carcinomas were as follows: 13/40 (32.5%) were complex, and 27/40 (67.5%) were simple carcinomas. Of the latter, 4/27 (14.8%) were tubular, 8/27 (29.6%) were tubulopapillary, 11/27 (40.7%) solid, and 4/27 (14.8%) anaplastic carcinomas. According to the histological grading system, 16/40 (40%) canine mammary carcinomas were classified as well-differentiated carcinomas (WDC), 13/40 (32.5%) as moderately differentiated carcinomas (MDC), and 11/40 (27.5%) as poorly differentiated carcinomas (PDC). The mean Mitotic Index was 11.4/mitoses per 10 HPF (median: 8.5), and 29 of the 40 (72.5%) canine mammary carcinomas were locally invasive while 11/40 (27.5%) exhibited lymphatic invasion or lymph node metastases. Twenty-five of the 40 (62.5%) dog bearing carcinomas were still alive at the end of the 2-years follow-up period, while 15/40 (37.5%) had died due to tumor-related causes. In cats, all the tumors (100%) were classified as carcinomas. Sixteen of the 30 queens (53.3%) had a tubulopapillary carcinoma, while 14 (46.7%) a solid carcinoma. The Mills classification showed that eight tumors were grade I (26.6%), 13 were Grade II (43.3%), and 9 were Grade III (30%). The mean MI was 22 mitoses/10 HPF (median: 19.5), and 8/30 carcinomas (26.7%) were only locally invasive, while 22/30 (73.3%) showed lymphatic vessel invasion. Seventeen queens (56.7%) had died for tumor-related causes, while 13 (43.3%) were still alive at the end of the 2-years follow-up period.

### 3.2. Immunohistochemistry

**Figure 2:**
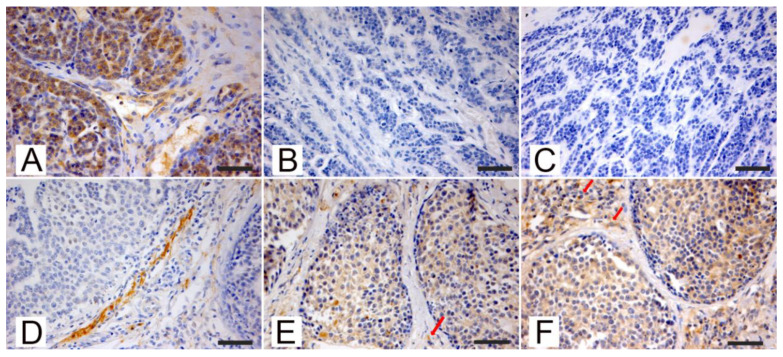
Canine mammary carcinomas, examples of positive phosphatase and tensin homolog (PTEN; (**A**,**D**)), pospho-AKT (p-AKT; (**B**,**E**)), and RICTOR (**C**,**F**) immunohistochemical expression. (**A**–**C**) Simple tubular CMC PTEN positive and p-AKT and RICTOR negative. (**A**) PTEN expression in cancer cells and in surrounding stromal cells, loss of p-AKT (**B**) and RICTOR (**C**) expression and in cancer cells. Streptavidin-biotin-peroxidase method, hematoxylin counterstain. Scale bar 50 micron. (**D**–**F**) Simple solid CMC PTEN negative and p-AKT and RICTOR positive. (**D**) Loss of PTEN expression in cancer cells, while surrounding stromal cells were PTEN-positive, p-AKT expression (**E**) and RICTOR expression (**F**) in cancer cells, scattered stromal cells were antigens positives (arrows). Streptavidin-biotin-peroxidase method, hematoxylin counterstain. Scale bar 50 micron.

The results of the immunohistochemistry investigations performed on CVMTs are presented in Figure 2.

**Figure 3:**
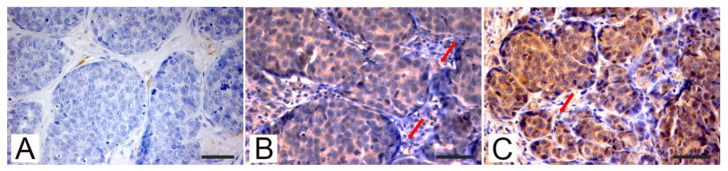
Feline mammary carcinomas, examples of positive phosphatase and tensin homolog (PTEN; (**A**)), pospho-AKT (p-AKT; (**B**)), and RICTOR (**C**) immunohistochemical expression. (**A**–**C**) Solid FMC PTEN negative and p-AKT and RICTOR positive. (**A**) Loss of PTEN expression in cancer cells, while surrounding stromal cells were PTEN-positive, p-AKT expression (**B**) and high RICTOR expression in cancer cells (**C**), scattered stromal cells were p-AKT and RICTOR positives (arrows). Streptavidin-biotin-peroxidase method, hematoxylin counterstain. Scale bar 50 micron.

Figure 3 shows the results of immunohistochemical investigations performed on feline mammary tissues.

In normal renal tissue sections used as controls, PTEN was almost exclusively expressed in proximal tubuli that can be histologically identified from distal tubuli by their smaller lumina and taller epithelial lining the lumen. In mammary tumor sections from both species, PTEN staining was mainly located in the cytoplasm and lesser in nuclei. In the majority of positive tumors, PTEN showed a homogeneous intensity pattern throughout the sample. However, some tumors were characterized by heterogeneous staining with a diffuse mild to moderate intensity and only strong focal positivity (Figure 1A). Stromal cells supporting neoplastic tissue often showed a strong positivity to PTEN IHC (Figure 1B and Figure 2A). P-AKT (Figure 1E and Figure 2B) and Rictor (Figure 1F and Figure 2C) staining were located both in the cytoplasm and the positive cells’ nucleus. Only the cytoplasmic signal was considered to assess the scoring of the positivity. Different from what was observed in PTEN immunostained samples, stromal cells were often negative or exhibited a mild nuclear or perinuclear signal Figure 1E,F and Figure 2B,C).

IHC results are summarized in Table 1. All the canine mammary adenomas (100%) and 25/40 CMCs (62.5%) were PTEN-positive. No canine adenoma (0%) and 15/40 (37.5%) CMCs were positive for p-AKT expression. Rictor was positive in 1/10 (10%) canine adenomas and 24/40 (60%) CMCs.

PTEN expression was inversely correlated with the neoplasm’s malignancy being more expressed in adenomas than in canine carcinomas (*p* = 0.002), while p-AKT and Rictor expression were statistically associated to CMCs (*p* = 0.021 and *p* = 0.005, respectively). Considering CMCs, PTEN expression was inversely correlated with p-AKT (*p* = 0.0001) and Rictor expression (*p* = 0.027). A positive statistical correlation was observed between p-AKT and Rictor expression in all CMTs (*p* = 0.0049) and carcinomas (*p* = 0.027). In queens, PTEN expression was inversely correlated with p-AKT expression (*p* < 0.0001), while for Rictor, even if 21/23 PTEN-negative FMVs were Rictor-positive, statistical significance was not achieved. Moreover, in the feline species, a strong positive statistical correlation was observed between p-AKT and Rictor expression (*p* = 0.0003).

The association between the immunohistochemical results and clinico-histopathological features, including histological subtype, lymphatic invasion, mitotic index, histological grading, and outcome of the neoplastic disease subjects, are summarized in Table 2 for the bitches and Table 3 for the queens.

As shown in Table 2, PTEN positively correlated with complex type carcinomas (*p* = 0.007), absence of lymphatic or lymph node invasion (*p =* 0.005), lower mitotic index (*p =* 0.009), WDC (*p =* 0.029) and better survival (*p =* 0.001). P-AKT was positively correlated with simple type carcinomas (*p =* 0.045), the lack of tubular structures (*p =* 0.031), lymphatic invasion (*p =* 0.001), and a poorer survival (*p =* 0.001). Rictor showed a positive correlation with the presence of lymphatic invasion (*p =* 0.001) and a poorer survival (*p =* 0.05).

As shown in Table 3, in FMTs, PTEN was positive in 7/30 (23.3%), p-AKT in 24/30 (80%), and Rictor in 20/30 (66.7%) carcinomas. The chi-square test showed that PTEN inversely correlated with p-AKT (*p =* 0.0001) and Rictor expression (*p =* 0.015). A highly significant positive correlation was observed between p-AKT and Rictor expression in feline carcinomas (*p =* 0.0001). In Table 3, the relations between the three markers and clinico-pathological features of FMCs are reported. PTEN positively correlated with the tubuli formation (*p =* 0.049), the absence of lymphatic invasion (*p =* 0.037), a lower mitotic index (*p =* 0.031), and better survival (*p =* 0.01). Furthermore, PTEN was more expressed in well-differentiated carcinomas than in moderately and poorly differentiated ones, even if this data was not statistically significant. P-AKT was positively correlated to poorer survival (*p =* 0.002). Even if this association was not statistically significant, P-AKT was also more expressed in tumors with lymphatic invasion. Rictor positively correlated with shorter survival (*p* = 0.001), and no other trends were evident from statistical analysis.

**Figure 4:**
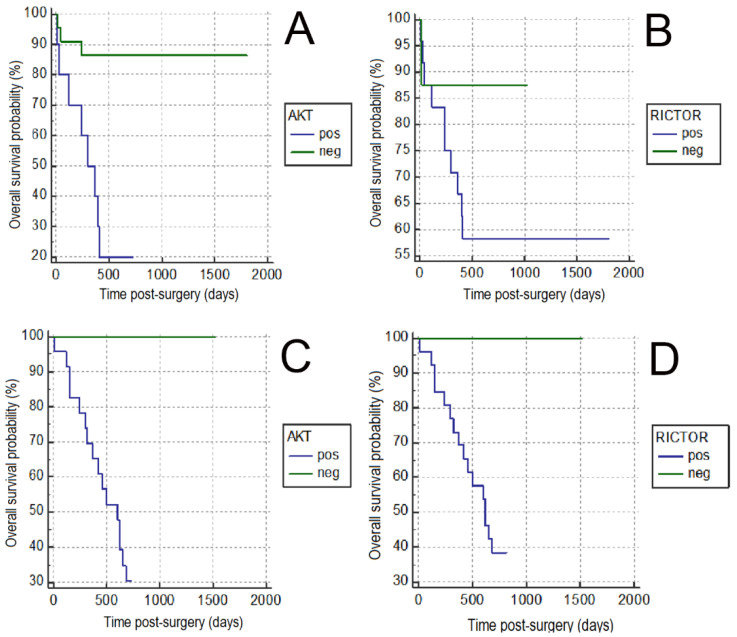
Kaplan-Meier curves depicting the overall survival of 40 bitches and 30 queens with invasive mammary carcinomas. (**A**) Overall survival curves for bitches bearing phosphoAKT (p-AKT)-negative and –positive CMCs. P-AKT positive tumors were associated with a poorer prognosis than negative CMCs (*p* = 0.001). (**B**) Overall survival curves for bitches bearing RICTOR-negative and –positive CMCs. RICTOR positive tumors were associated with a poorer prognosis than negative CMCs (*p* = 0.05). (**C**) Overall survival curves for queens bearing p-AKT-negative and –positive FMCs. P-AKT -positive tumors were associated with a poorer prognosis than negative FMCs (*p* = 0.002). (**D**) Overall survival curves for queens bearing RICTOR-negative and –positive FMCs. RICTOR positive tumors were associated with a poorer prognosis (*p* = 0.001).

Cancer-specific survival analysis is presented in Figure 4. In both species, cancer-specific survival analysis confirmed the significant correlation between p-Akt- and Rictor-positive expression and cancer-specific overall survival. In particular, bitches bearing tumors p-Akt-positive had a poor prognosis with a mean cancer-specific survival time of 233 ± 163 days and a median survival time of 270 days (*p* = 0.001), and in animals bearing Rictor-positive carcinomas, the mean cancer-specific overall survival was 216 ± 155 days with a median survival time of 240 days (*p* = 0.05). Additionally, queens bearing p-Akt-positive carcinomas had a poor prognosis with a mean cancer-specific survival time of 388 ± 214 days and a median survival time of 310 days (*p* = 0.002). In animals bearing Rictor-positive tumors, the mean cancer-specific overall survival was 388 ± 214 days, with a median survival time of 395 days (*p* = 0.001).

## 4. Discussion

Several pieces of evidence confirm the PTEN/AKT pathway involvement in human malignancies [3,5]. The activation of such proteins leads to various downstream biological effects that promote tumor growth, such as inhibition of the apoptosis [3], stimulation of cell cycle and proliferation [3], neoangiogenesis [5], production of metalloproteinases [3], and inhibition of cell to cell adhesion [57]. This pathway’s dysregulation has been often associated with several cancers in humans [3,5] and small animal medicine [17,18,21,22].

PTEN expression was lost or reduced in 36% of canine and 77% of the feline mammary carcinomas in our study. In CMTs, PTEN was significantly more expressed in adenomas than in carcinomas, in agreement with our previous findings [21] and those of Qiu and colleagues [23]. The same comparison could not be performed for FMTs due to the absence of mammary adenomas in our population. Furthermore, all the canine adenomas were PTEN-positive confirms that PTEN plays a decisive role in limiting tumor progression, as widely demonstrated in human medicine [58,59,60]. Considering tumor features, in dogs PTEN correlated with complex and well-differentiated carcinomas, the absence of lymphatic vessel invasion, lower MI, and a longer survival period. In cats, PTEN was correlated to tubulopapillary carcinomas, absence of lymphatic vessel invasion, lower MI, and longer survival. These findings strengthen the PTEN association with better tumor behavior, as previously reported in CMTs and FMTs [21]. This study confirmed a previous report in which there is a significant association between PTEN loss and a significant reduction of overall survival, confirming the role of PTEN as tumor-suppressor protein even in the canine species [20]. PTEN reduction and loss have also been reported in human cancers [5,6,13,58] and in canine melanoma [16], hemangiosarcoma [17], osteosarcoma [18], and in CMTs and FMTs [2,22].

AKT over-production has been observed in a large number of human malignancies [21,27,29,31,32,61,62] in FMTs [22] and in canine hemangiosarcoma’s [17,33], osteosarcomas [18], melanomas [15], neuroepithelial [37] and mast cell tumors [41]. In our study, p-AKT expression was detected in 38% of canine and 80% of feline mammary carcinomas, while no canine adenoma was p-AKT-positive. Furthermore, p-AKT expression was higher in canine carcinomas than in adenomas. No data are available in the literature about p-AKT expression in CMTs. In human breast carcinoma, p-AKT seems to be more expressed than in CMTs, with the percentage of positivity ranging between 54% and 76% [30,63]. The latter percentage is similar to what was found in our feline population and a previous study in FMTs [22]. In dogs, p-AKT expression was associated with simple carcinomas, lymphatic invasion, absence of tubular structures, and poorer prognosis. In cats, p-AKT was correlated to poorer survival times, as previously reported in human breast cancer and FMTs [22]. These findings confirm the decisive AKT role within this pathway and in tumor progression, particularly for what concerns its form phosphorylated at Serine473. Our data demonstrated that the fully activated AKT form is the p-AKT Serine473, as previously reported [25].

The mTORC2 is responsible for AKT phosphorylation, and it is thus considered the AKT activator [25]. Among its different components, Rictor is considered the most important [64] and its expression has been linked to breast [43,65], prostatic [66], ovarian [67], colorectal [48], cancer and gliomas [50]. In our study, Rictor was expressed in only one canine mammary adenoma (10%), in 60% of the canine carcinomas, and 67% of feline mammary carcinomas. In dogs, Rictor expression was correlated to mammary carcinomas and lymphatic invasion. In FMTs, Rictor was associated with a poorer prognosis. To the best of our knowledge, this is the first study investigating Rictor’s implication in CMTs and FMTs. A similar percentage of Rictor positivity (64%) was observed in humans in humans in breast cancer by Zhang and colleagues [65]. Our data suggest that Rictor plays an oncogenic role in CMTs and FMTs, even if its action seems less incisive than p-AKT. Particularly in dogs, p-AKT was associated with more aggressive tumor features than Rictor, which confirms its key role in the PTEN/AKT pathway.

However, this study’s main aim was to investigate the mechanisms responsible for the development of this signaling system. To achieve this purpose, we compared PTEN, p-AKT, and Rictor expression between them. Statistical analysis Chi-square test showed a strong negative correlation between PTEN and p-AKT expression both in CMTs and FMTs. Briefly, when PTEN is normally expressed, AKT levels are controlled or completely inhibited [3]. When PTEN expression is reduced or lost, AKT can be continuously produced and phosphorylated at Serine473, leading to a continued increase of p-AKT cellular levels [3,68]. The association between PTEN reduction and AKT increase has been previously reported even in canine osteosarcoma cell lines [18]. In canine hemangiosarcoma’s instead, AKT was expressed by a small number of tumors, and its expression was not associated with PTEN loss of expression [17]. PTEN inactivation can be due to different causes, such as somatic and germline mutations [69], haploinsufficiency [70], loss of the heterozygosity [71], hypermethylation [72], deletion [73], and protein phosphorylation [74]. All these alterations of PTEN expression promote AKT activation and tumor behavior worsening [3]. Moreover, germline mutations in PTEN gene are also the cause of the Cowden syndrome, an autosomal dominant disorder linked to several benign and malignant diseases [75]. To summarize, PTEN expression reduction is linked to the increase of the tumor behavior aggressiveness as a consequence of the uncontrolled activation of AKT.

P-AKT was positively correlated to Rictor expression both in CMTs and FMTS. This finding seems to confirm the tight relation between these two proteins, with the complex mTORC2 that determines the full activation of AKT throughout its phosphorylation at Serine473 [63]. However, 4 FMTs and in 1 CMT were p-AKT -positive and Rictor-negative. This data disagreed with the theory that considers Rictor necessary for AKT phosphorylation. In fact, in these five tumors, AKT was phosphorylated even if the same neoplasms did not express Rictor. A probable explanation of this finding is that Rictor is the most important activator of AKT. However, several other molecules can play this role, such as integrin-linked kinase, MAPKAP kinase 2, DNA-dependent kinase, ataxia telangiectasia mutated, protein kinase C, and p21-activated kinase 1 and 2 [42]. This aspect further explains that Rictor oncogenic effects are less incisive than AKT ones and are strictly related to its downstream activation.

A negative correlation between PTEN and Rictor expression was also observed in CMTs and FMTs, even if this association was less strong than that observed between PTEN and p-AKT. No evidence is reported in the veterinary literature about these two proteins’ interaction or between PTEN and mTORC2. Authors speculate that Rictor expression does not depend on PTEN action and that this statistically negative correlation may be a coincidence caused by the common association of PTEN with less aggressive tumors and Rictor with more aggressive cancer.

Our data confirm the strong implication of the PTEN/AKT pathway in CMTs and FMTs progression. Furthermore, data about the correlation between the three markers showed that their roles within this system are similar to the ones reported in human medicine: PTEN is the main inhibitor [3], AKT plays the pivotal role, and Rictor (and its complex mTORC2) is one of the main activators of AKT [25]. The involvement of this pathway in CMTs and FMTs also has some prognostic and therapeutic implications. The strong association of PTEN expression with longer and of p-AKT with shorter survival periods could suggest a future use of anti-PTEN and anti-p-AKT IHC to evaluate CMTs and FMTs prognosis better. Furthermore, in human medicine, several AKT inhibitors are under investigation for the pharmacological treatment of various malignancies [7]. The evidence that p-AKT is the key-molecule of this vital pathway could provide the rationale for future studies of AKT inhibitors in CMTs and FMTs.

## 5. Conclusions

Our study demonstrated for the first time a close correlation between PTEN loss and negative evolution of FMCs and confirmed that the PTEN/AKT pathway plays an incisive role in CMTs and FMTs. Among the several components of this system, PTEN plays the most important tumor-suppressor role, AKT is the key-molecule responsible for tumor aggressiveness increase, and Rictor (and the mTORC2) is the most important AKT activator. To the best of our knowledge, this is the first study that comprehensively explores the PTEN/AKT pathway in CMTs and FMTs. Findings emerged from our study, and their prognostic and therapeutic implications provide a rationale for further studies focused on PTEN/AKT pathway involvement in canine and feline malignancies.

## Figures and Tables

**Table 1:** Immunohistochemical expression of PTEN, phospho-AKT (p-AKT), and Rictor in the canine and feline mammary tumors examined.

Tumors	PTEN		p-AKT		Rictor	
	Positive	%	Positive	%	Positive	%
DOG						
Adenomas	10/10 ^a^	100	0	0	1	10
Carcinomas	25/40	62.5	15/40 ^b^	37.5	24/40 ^c^	60
CAT						
Carcinomas	7/30	23.3	24/30	80	20/30	66.7

Fisher test analysis: ^a^ PTEN expression was significantly higher (*p* = 0.021) in canine mammary adenomas than in canine carcinomas. ^b^ p-AKT expression was significantly higher (*p* = 0.021) in canine mammary carcinomas than in canine adenomas. ^c^ Rictor expression was significantly higher (*p* = 0.005) in canine mammary carcinomas than in canine adenomas.

**Table 2:** Comparison between PTEN, posho-AKT (p-AKT), and Rictor immunohistochemical expression and canine carcinomas features. Tumor grading was performed according to Peña et al. [52], and all comparisons were conducted using the Fisher test.

*n* = 30	PTEN			p-AKT			Rictor		
	Pos	Neg	*p*	Pos	Neg	*p*	Pos	Neg	*p*
Histotype									
Tubular and tubulopapillary	6	10	0.049	12	4	0.464	10	6	0.604
Solid	1	13		12	2		10	4	
Lymphatic invasion									
Negative	4	4	0.037	5	3	0.148	4	4	0.243
Positive	3	19		19	3		16	6	
Mitotic index									
<Median	6	9	0.031	11	4	0.361	9	6	0.439
>Median	1	14		13	2		11	4	
Mills grade									
I	4	5	0.192	6	3	0.359	5	4	0.607
II	2	10		11	1		8	4	
III	1	8		7	2		7	2	
Overall survival			0.010			0.002			0.001
Alive	6	7		7	6		4	9	
Dead	1	16		17	0		16	1	

**Table 3:** Comparison between PTEN, pospho-AKT (p-AKT) and Rictor immunohistochemical expression and feline carcinomas features. Tumor grading was performed according to Mills et al. [53], and all comparisons were conducted using the Fisher test.

*n* = 40	PTEN			p-AKT			Rictor		
	Pos	Neg	*p*	Pos	Neg	*p*	Pos	Neg	*p*
Morphology									
Complex	12	1	0.007	2	11	0.045	8	5	0.890
Simple	13	14		13	14		16	11	
Histotype									
Tubular and Tubulopapillary	7	5	343	3	9	0.031	3	9	0.247
Solid and Anaplastic	6	9	0.	10	5		7	8	
Lymphatic invasion									
Negative	22	7	0.005	6	23	0.001	13	16	0.001
Positive	3	8		9	2		11	0	
Mitotic index									
<Median	16	4	0.009	5	15	0.102	15	5	0.053
>Median	9	11		10	10		9	11	
Peña grade									
WDC	14	2	0.029	3	13	0.124	8	8	0.218
MDC	6	7		6	7		7	6	
PDC	5	6		6	5		9	2	
Overall survival									
Alive	21	4	0.001	3	22	0.001	12	13	0.050
Dead	4	11		12	3		12	3	

## Abbreviations

CMA, canine mammary adenoma; CMC, canine mammary carcinoma; FMC, feline mammary carcinoma; p-AKT, pospho-AKT; IHC, immunohistochemistry; PTEN, phosphatase and tensin homolog.
